# Investigating the shared genetic architecture between frailty and insomnia

**DOI:** 10.3389/fnagi.2024.1358996

**Published:** 2024-02-15

**Authors:** Zhiwei Song, Wangyu Li, Yupeng Han, Yiya Xu, Yinzhou Wang

**Affiliations:** ^1^Department of Neurology, Fujian Provincial Hospital, Shengli Clinical Medical College of Fujian Medical University, Fuzhou, Fujian, China; ^2^Department of Pain Management, Fujian Provincial Hospital, Shengli Clinical Medical College of Fujian Medical University, Fuzhou, Fujian, China; ^3^Department of Anesthesiology, Fujian Provincial Hospital, Shengli Clinical Medical College of Fujian Medical University, Fuzhou, Fujian, China; ^4^Fujian Key Laboratory of Medical Analysis, Fujian Academy of Medical Sciences, Fuzhou, Fujian, China

**Keywords:** frailty, insomnia, shared genetic architecture, Mendelian randomization, causal relationship

## Abstract

**Background:**

The epidemiological association between frailty and insomnia is well established, yet the presence of a common genetic etiology is still uncertain. Further exploration is needed to ascertain the causal relationship between frailty and insomnia.

**Methods:**

Utilizing data obtained from genome-wide association studies (GWAS) summaries, we utilized the linkage disequilibrium score regression (LDSC) to determine the genetic correlation existing between frailty and insomnia. The determination of causality was achieved through the application of two-sample Mendelian randomization. We investigated the enrichment of single nucleotide polymorphism (SNP) at various tissue types utilizing stratified LD score regression (S-LDSC) and multimarker analysis of genome annotation (MAGMA). Common risk SNPs were identified using Multi-Trait Analysis of GWAS (MTAG) and Cross-Phenotype Association (CPASSOC). We further investigated the expression profiles of risk genes in tissues using Summary-data-based Mendelian randomization(SMR) based on pooled data, to explore potential functional genes.

**Results:**

Our findings indicated a significant genetic correlation between frailty and insomnia, highlighting SNPs sharing risk (rs34290943, rs10865954), with a pronounced correlation in the localized genomic region 3p21.31. Partitioned genetic analysis revealed 24 functional elements significantly associated with both frailty and insomnia. Furthermore, mendelian randomization revealed a causal connection between frailty and insomnia. The genetic correlation between frailty and insomnia showed enrichment in 11 brain regions (S-LDSC) and 9 brain regions (MAGMA), where four functional genes (RMB6, MST1R, RF123, and FAM212A) were identified.

**Conclusion:**

This study suggests the existence of a genetic correlation and common risk genes between frailty and insomnia, contributing to a deeper comprehension of their pathogenesis and assists in identifying potential therapeutic targets.

## 1 Background

Frailty, a common geriatric syndrome, is defined as an age-related state characterized by impaired biological reserves, a reduced ability to maintain physiologic homeostasis, and an increased vulnerability to adverse outcomes (Dent et al., 2019). With the increasing rate of population aging, concerns about frailty and its impact on the global health burden are intensifying. Frailty is commonly assessed using the frailty index (FI) (Atkins et al., 2021), defined as the proportion of accumulated health deficits (signs, symptoms, dysfunctions, and laboratory abnormalities) over a lifetime. FI is better at identifying frailty in its low to mid-range spectrum compared to the frailty phenotype (FP), rendering it more sensitive in younger individuals. Additionally, FI is considered predictive of the risk of falls, fractures, disability, and death (Shi et al., 2021). Insomnia, a prevalent sleep disorder, affects approximately 10% of adults chronically and an additional 20% experience occasional insomnia symptoms (Perlis et al., 2022). Insomnia prevalence is higher among women, the elderly, and individuals facing economic hardship. Growing evidence suggests that individuals with insomnia may experience multiple somatic and psychological disorders, impaired quality of life, and higher all-cause mortality rates (Ge et al., 2019). Evidence indicates that insomnia can result in sleep dysfunction, fatigue, unsteady gait, and decreased physical activity, all factors that elevate the risk of frailty (Fan et al., 2022). Furthermore, the variety of psychological and somatic disorders induced by insomnia may heighten vulnerability to frailty. Recently, there has been an increasing body of evidence suggesting the coexistence of frailty and insomnia. A recent meta-analysis encompassing 12 observational studies (Pourmotabbed et al., 2020) suggests an association between sleep disturbances and frailty. However, prior studies exploring the potential association between frailty and insomnia have produced inconsistent results (Nemoto et al., 2021; Tang et al., 2021; Wen et al., 2023), making it challenging to establish causality.

Frailty and insomnia both possess a substantial genetic basis. Studies have shown that frailty has a genetic foundation, with estimates of heritability varying from 30 to 45% (Livshits et al., 2018). A recent extensive GWAS study identified 202 common genetic loci associated with insomnia (Jansen et al., 2019). Few studies to date have explored the link between frailty and insomnia at a genetic level, and it remains unclear as to whether and how their genetic structures overlap. Based on the established link between frailty and insomnia in epidemiological studies, we hypothesize that frailty and insomnia could share a common genetic structure and that their relationship might entail a causal connection. Based on this hypothesis, it is anticipated that this study will yield new insights into the pathophysiological mechanisms underlying the co-morbidity of frailty and insomnia, and identify new therapeutic targets for the two diseases, whether occurring individually or in combination, thereby contributing to healthier, more independent aging.

Cross-trait analysis (Zhu et al., 2021) employs various meta-analytic methods to integrate summary statistics of distinct yet potentially related traits. This approach aims to identify specific loci with shared associations within a meta-analytic framework. It contributes significantly to the comprehension of the genetic architecture of complex traits, facilitating the exploration of genetic sharing, trait interactions, and potential biological mechanisms. Multi-trait analysis of GWAS (MTAG) (Turley et al., 2018) represents a methodology for concurrent multi-phenotypic analyses, utilizing GWAS generalized new data. This approach enables the leveraging of information from associated phenotypes to augment the statistical power of tests for target phenotypes, offering an advantage over single-phenotype GWAS. Cross-Phenotype Association (CPASSOC) (Zhu et al., 2021) employs aggregated single SNP-trait associations from GWAS to ascertain which variants are associated with at least one trait. It exhibits several benefits in identifying cross-phenotypic associations, including the ability to accommodate opposite risk effects and various types of phenotypic traits. Cross-trait analyses are currently being applied in the field of neurology. For example, Tian et al. (2023) have demonstrated a shared genetic framework between amyotrophic lateral sclerosis and Parkinson’s disease, encompassing 9 single-nucleotide polymorphisms, 3 risk loci, and 7 genes. These genes are linked to neuronal projection development pathways. In another study, Zeng et al. (2023) discovered a notable positive genetic link between body mass index and multiple sclerosis, indicated by 39 common risk SNPs. Such results provide new perspectives on the mechanisms driving their co-morbidities and guide upcoming treatment strategies.

Mendelian randomization has been utilized in studies of frailty and insomnia, leveraging its ability to mitigate the effects of confounders and diminish reverse causal inference (Emdin et al., 2017). For instance, Deng et al. (2023) demonstrated a bidirectional causal relationship between frailty and depression from a genetic standpoint via Mendelian randomization. This suggests that routine frailty screening for depressed patients is necessary, and treating depression may also moderately reduce the risk of frailty. Utilizing Mendelian randomization, researchers have determined that various sleep characteristics (short sleep, long sleep, continuous sleep duration) not only are associated with but also causally influence the prognosis of ischaemic stroke, potentially serving as a therapeutic target to enhance stroke prognosis (Zhang et al., 2023).

**Figure 1:**
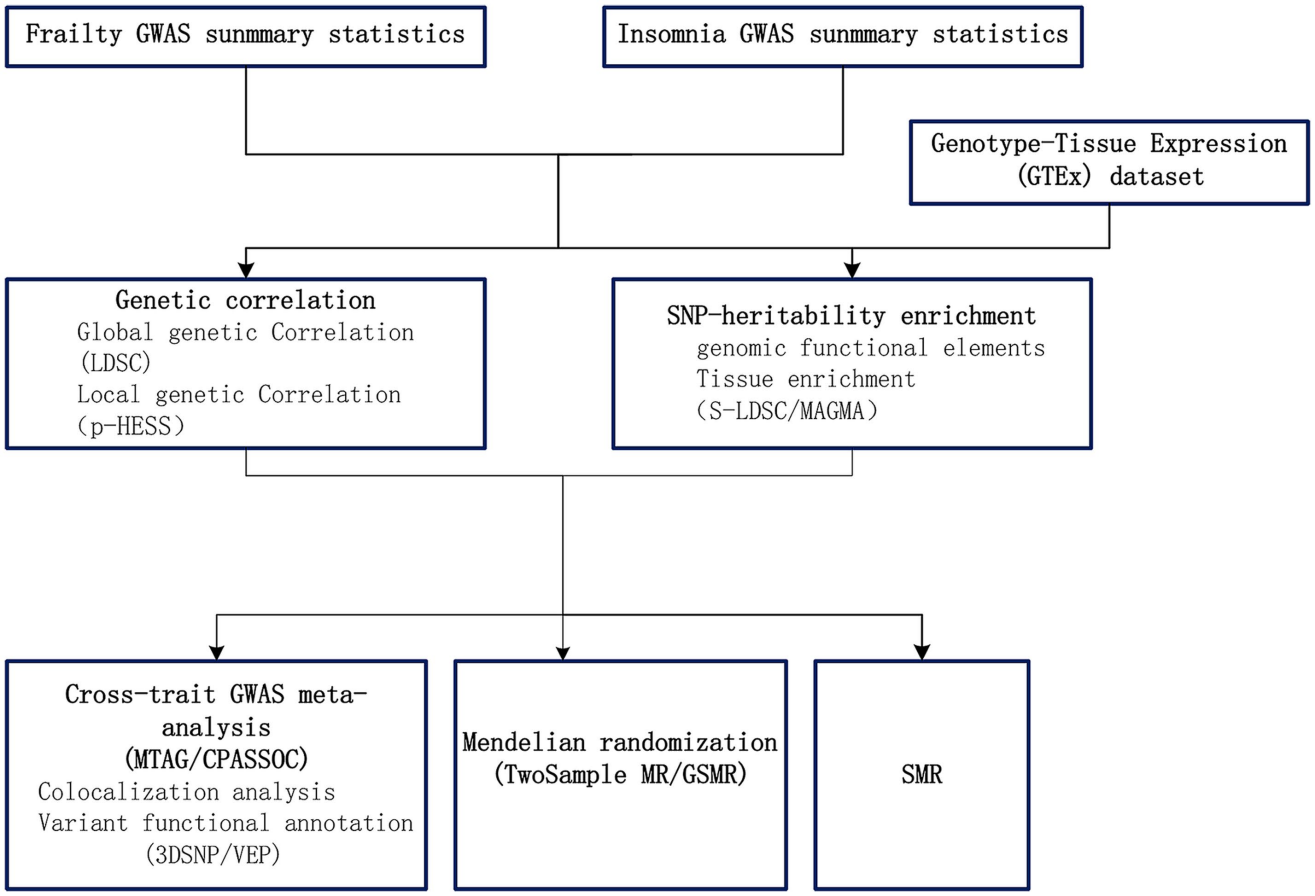
Overview of analytical strategy performed in the study.

In our research, we explored the genetic links and the possibility of a causal connection between frailty and insomnia, using extensive GWAS data. Initially, to comprehend the shared genetic bases of frailty and insomnia, we established the genetic correlations and local genetic correlations. Subsequently, we utilized cross-trait GWAS meta-analyses for identifying common SNPs. Moreover, we implemented two-sample Mendelian randomization analyses to examine the causative link between frailty and insomnia. Furthermore, our study involved analyzing the tissue-specific enrichment of genetic associations linking frailty and insomnia. Summary data-based Mendelian randomization (SMR) (Zhu et al., 2016) utilizing pooled data, is a methodology that merges summary data from GWAS with data from expression quantitative trait loci (eQTL) studies in order to identify genes whose expression levels are linked to complex traits owing to pleiotropy. Utilizing SMR, we pinpointed genes with shared functionality between frailty and insomnia. The STROBE-MR guidelines (Skrivankova et al., 2021a,b) were followed throughout the course of the study. Figure 1 presents a flowchart depicting our analytical strategy.

## 2 Materials and methods

### 2.1 Study samples

The genesis of the GWAS data on frailty can be traced to a study led by Atkins et al. (2021). This research involved a GWAS on frailty indices among participants from the British Biobank of European descent (*n* = 164,610, aged between 60 and 70) and those from the Swedish TwinGene project (*n* = 10,616, aged 41–87). Calculations for the FI were based on 49 or 44 self-reported elements, including symptoms, disabilities, and diagnosed illnesses. The complete GWAS summary statistics are available for download from the GWAS catalog (project number: GCST90020053). Meanwhile, the summary statistics for insomnia were derived from a GWAS using UK Biobank data (project number: ukb-b-3957), which included 462,341 participants.

### 2.2 Heritability and genetic correlation

Utilizing GWAS summary statistics and LD scores from European lineage reference data in the 1,000 Genomes Project, we applied the linkage disequilibrium score regression (LDSC) (Bulik-Sullivan et al., 2015) to ascertain the heritability of frailty and insomnia, as well as to determine the genetic correlation between these traits. Heritabilities of SNPs for frailty and insomnia were measured using S-LDSC (Finucane et al., 2015) in combination with the baseline LD model (Gazal et al., 2017). This baseline LD model approach distinctly determines SNP heritabilities based on continuous, rather than binary, annotation sets. The use of S-LDSC (Finucane et al., 2015) facilitates the analysis of the genetic structure of complex traits by dividing heritability among various genomic annotations. Following this, bivariate LDSC with unconstrained intercepts was utilized to gauge genetic correlations between frailty and insomnia (rg). Considering the population overlap in the GWAS data for frailty and insomnia, an LDSC with constrained intercepts was also conducted as part of a sensitivity analysis.

### 2.3 Local genetic correlation analysis

Heritability Estimation from Summary Statistics (ρ-HESS) (Shi et al., 2017) is employed for estimating local SNP heritability and genetic correlation from summary statistics. Local genetic correlations were estimated using ρ-HESS to determine whether frailty and insomnia are genetically correlated in locally independent regions of the genome. Subsequently, the local SNP heritabilities and genetic correlations between the two traits were calculated, employing the 1,000 Genomes Project data provided on the ρ-HESS webpage as a reference. Subsequently, the Bonferroni correction method was utilized to adjust for multiple testing, considering the factor of 0.05 divided by the number of regions.

### 2.4 Partitioned heritability

Our study provided further insight into the genetic correlation between frailty and insomnia through the analysis of various genomic functional elements via S-LDSC (Okamura et al., 2016), which operates by categorizing SNPs into functional groups and subsequently calculating LD scores for each categorized SNP. The calculated LD scores were subsequently utilized to estimate the genetic correlation within each functional category. Consequently, this approach enabled the estimation of genetic correlations across over 30 functional components, thereby elucidating the contribution of diverse components to the overarching genetic correlation between frailty and insomnia.

### 2.5 Tissue specific enrichment of SNP heritability

#### 2.5.1 S-LDSC

We conducted a GTEx tissue enrichment analysis using S-LDSC to determine the tissues most strongly associated with shared genes (Finucane et al., 2015). GTEx (version 8) offers insights into 53 distinct tissue types, encompassing data on SNP mutations linked to quantitative traits of gene expression across various tissues.

#### 2.5.2 MAGMA

For a sensitivity analysis of S-LDSC, tissue-specific enrichment and gene set enrichment analyses were performed using MAGMA (de Leeuw et al., 2015). The process began with a gene-level association analysis to assess the relationship between genes and phenotypes, utilizing the *p*-values of SNPs in proximity to the genes in question. Subsequently, gene set enrichment analyses were performed using the designated gene set of FUMA (MSigDB_20231Hs_MAGMA). Finally, for the assessment of the tissue specificity of the phenotype, MAGMA gene characterization was employed. This analysis investigated the relationship between tissue-specific gene expression profiles and disease gene associations, utilizing data from GTEx v8 (gtex_v8_ts_avg_log2TPM), which encompasses information from 54 distinct tissues.

### 2.6 Cross-trait GWAS meta-analysis

In order to pinpoint shared risk SNPs associated with frailty and insomnia, cross-trait meta-analyses were conducted employing MTAG (Turley et al., 2018) and CPASSOC (Zhu et al., 2021). In MTAG analyses, the estimation of SNP effects for each trait can be enhanced by including related, distinct traits (Yoshida and Yáñez, 2021). Additionally, a paired cross-trait meta-analysis was executed using CPASSOC to consider the variance in heritability of the two phenotypes. CPASSOC operates on the assumption that cross-trait heterogeneity effects exist, and it calculates cross-trait statistical heterogeneity (SHet) and *p*-values via a meta-analysis weighted by sample size. SHom is typified as a fixed-effects meta-analysis approach with diminished efficacy when faced with between-study heterogeneity. In cases of heterogeneous effects, SHet serves as an expansion of SHom, providing enhanced statistical stability and power. We utilized SHet to combine summary statistics for frailty and insomnia.

SNPs of significance were identified based on their notable associations with both phenotypes, characterized by a *p*-value less than 5 × 10^-8 in both MTAG and CPASSOC analyses. The summary statistics for frailty and insomnia were merged using SHet, followed by clustering them using parameters in PLINK (1.9). Independent SNPs, significantly associated with the phenotype, were identified by applying specific parameters through PLINK’s “clustering” function: -clump-p1 5e-8 -clump-p2 1e-5 -clump-r2 0.2 - clump-kb 500.

### 2.7 Mendelian randomization

To investigate the potential causal relationship between frailty and insomnia, the “TwoSampleMR” and “GSMR” R packages were employed to analyze associations suggestive of causality (*p* < 0.05). Mendelian stochastic analyses were conducted using six principal MR methods, namely MR-Egger (Burgess and Thompson, 2017), inverse variance weighting (IVW) (Burgess et al., 2017), weighted median, weighted mode, simple mode, and GSMR, with varying assumptions regarding the level of multinomiality. Evaluations of horizontal pleiotropy and heterogeneity were performed using the MR-Egger intercept test and Cochran’s Q statistic. MR-PRESSO (Verbanck et al., 2018) were utilized to identify pleiotropy and outliers. Variant selection was predicated on three fundamental assumptions: that the variants are (1) exposure-related, (2) not influenced by confounders, and (3) devoid of a direct impact on the outcomes.

### 2.8 Summary-data-based Mendelian randomization

SMR (Krishnamoorthy et al., 2023) uses genetic variation as an instrumental variable for estimating the impact of a gene’s expression level on the phenotype. To identify shared functional genes in tissue enrichment, both the Benjamini-Hochberg FDR test and the HEIDI-outlier test (Yang et al., 2021) were utilized.

## 3 Results

### 3.1 Genetic correlations

The liability-scale SNP heritability (without constrained intercept) was determined to be 11.69% (95% CI = 11.68–11.70%) for frailty and 6.65% (95% CI = 6.65–6.65%) for insomnia, with a significantly positive genetic correlation between frailty and insomnia (rg = 1.14, *p* = 0.00). Additionally, LDSC with constrained intercepts was conducted (rg = 0.61, *p* = 4.63E-189), and the findings continued to be significant (Supplementary Table S1).

### 3.2 Local genetic correlations

After applying multiple corrections, the ρ-HESS method was employed to determine a significant local genetic correlation between frailty and insomnia. A significant local correlation was identified in a singular region between frailty and insomnia at 3p21.31 (chr3:47727212 … 0.49316972) (*p* = 9.45e-06) (Supplementary Figure S1; Supplementary Table S2).

### 3.3 Partitioned genetic correlations

**Figure 2:**
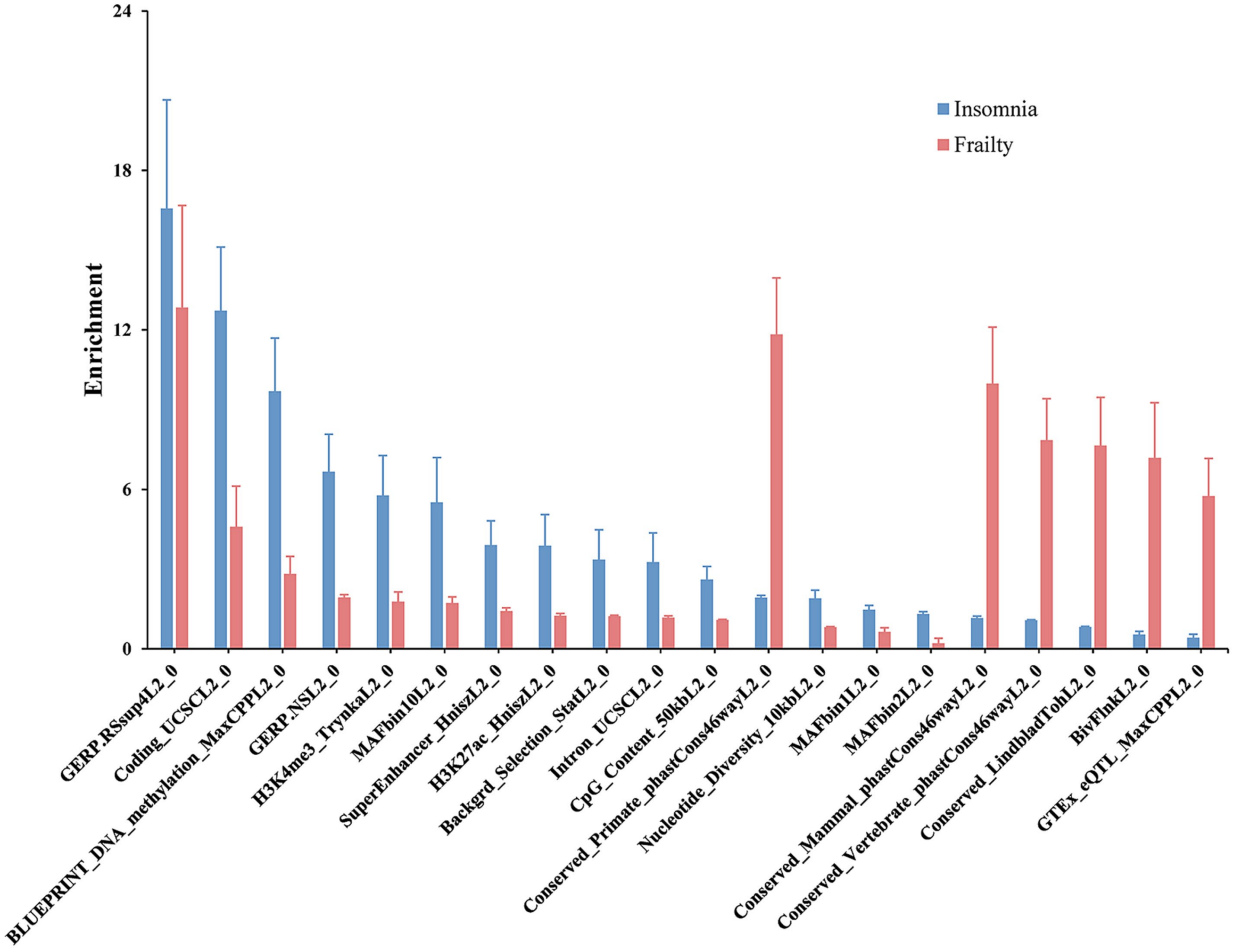
Partitioned genetic correlation between frailty and insomnia. The display highlighted genomic functional elements common to both frailty and insomnia. The vertical axis depicted estimates of enrichment, and error bars indicated the standard error of enrichment.

SNPs partially genetically related to frailty demonstrated enrichment in 31 of 98 functional categories, with the leading category being GERP.RSsup4L2_0 (enrichment = 12.8, *p* = 0.00). SNPs associated with insomnia showed enrichment in 36 of 97 functional categories, notably led by GERP.RSsup4L2_0 (enrichment = 16.6, *p* = 0.00). Twenty-four functional categories exhibited significant associations with both frailty and insomnia (Figure 2; Supplementary Table S3).

### 3.4 Cross-trait GWAS meta-analysis

Cross-trait analyses were conducted using both MTAG and CPASSO, resulting in 124 shared independent SNPs achieving genome-wide significance (Supplementary Table S4). In the screening of SNPs at 3p21.31, after excluding SNPs that were only significant in GWAS for frailty or insomnia, or those in linkage disequilibrium (LD *r*^2^ ≥ 0.02) with previously significant SNPs, two SNPs were identified as being associated with both frailty and insomnia (Table 1). The rs34290943 SNP was mapped to the DALRD3 gene, and locus rs10865954 to the MIR191 gene.

**Table 1:** Shared risk regions in different analyses.

SNP	CHR	BP	Effect allele	Non-effect allele	Mtag_pval_frailty	Mtag_pval_insomnia	CPASSOC *p*-value	ρ-HESS *p*-value
rs34290943	3	49,161,660	C	T	5.81E-10	5.63E-10	7.55E-09	9.45E-06
rs10865954	3	49,211,989	C	T	4.73E-12	3.53E-12	5.38E-13	9.45E-06

### 3.5 Mendelian randomization

**Figure 3:**
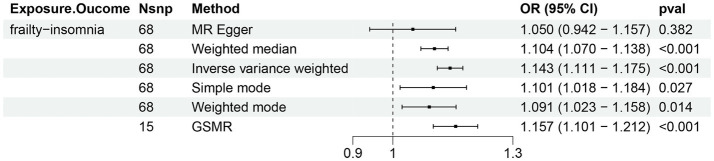
Bi-directional Mendelian randomization (MR) analyses between frailty and insomnia. (Causal effect of frailty on insomnia). Estimates and 95% confidence intervals (CI) are shown using square plots and error bars.

Two-sample Mendelian randomization was conducted using loci significantly associated with either the frailty or insomnia phenotype as instrumental variables (*F* > 10). Given the genetic commonalities identified in the preceding section, causality may arise from instrumental variables exhibiting pleiotropic effects. Upon exclusion of potentially biased polytropic instrumental variables, MR was reanalyzed, revealing a strong causal relationship (OR 1.14, *p* < 0.001) (Figure 3; Supplementary Table S5). The Egger intercept did not deviate from 0, indicating that the remaining instrumental variables (IVs) were not affected by polytropy. Conversely, the causal relationship between insomnia and frailty was found to be unstable. Insomnia potentially played a role in elevating the risk of frailty (OR 1.98, *p* = 0.00), yet the presence of horizontal pleiotropy could not be discounted (Egger intercept = 0.004, *p* = 0.007) (Supplementary Table S6).

### 3.6 Tissue-level SNP heritability enrichment

The enrichment of SNP heritability for frailty and insomnia at the tissue level was evaluated using S-LDSC analysis, which involved GTEx data across different tissues. Tissues exhibiting significant SNP heritability enrichment for frailty and insomnia, predominantly located in the brain, were identified. Specifically, SNPs associated with frailty showed notable enrichment in 11 different brain regions, primarily in the anterior cingulate cortex (BA24) and cerebellar hemispheres.

**Figure 4:**
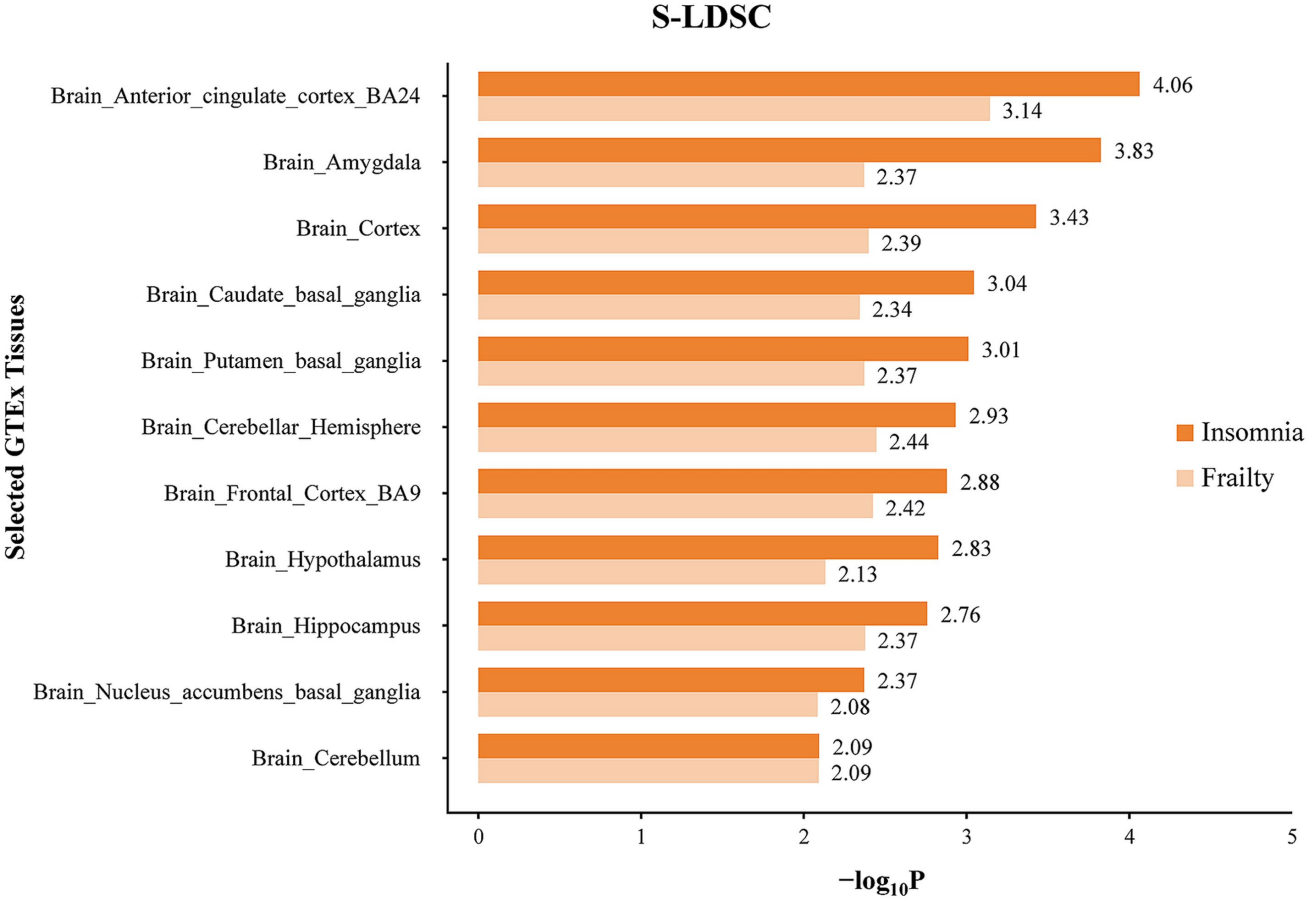
Tissue specific enrichment of SNP heritability for frailty and insomnia through S-LDSC. The x-axis represents negative log10 *p*-values of enrichment for each individual test.

**Figure 5:**
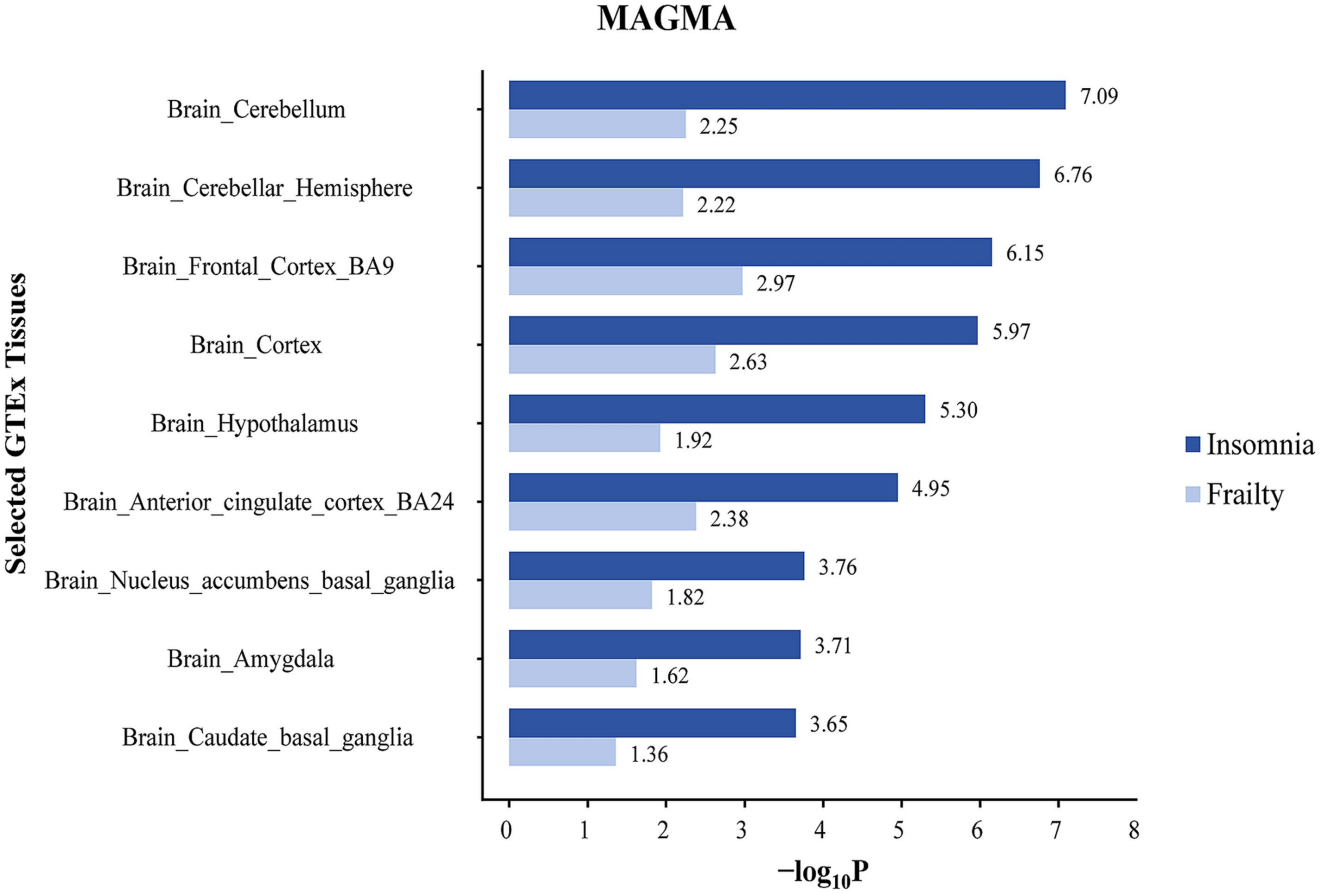
Tissue specific enrichment of SNP heritability for frailty and insomnia through MAGMA. The x-axis represents negative log10 *p*-values of enrichment for each individual test.

In the case of insomnia, distinct enrichment of SNPs was noted in 19 different brain regions, predominantly concentrated in the anterior cingulate cortex (BA24) and the cerebral amygdala. SNPs co-associated with frailty and insomnia exhibited enrichment in 11 brain regions, mainly in the anterior cingulate cortex (BA24) and cerebral amygdala (Figure 4; Supplementary Table S7). Furthermore, MAGMA tissue-specific analyses revealed that SNPs linked to frailty showed specific enrichment in 11 distinct brain regions, whereas for insomnia, such enrichment was observed in 14 different brain areas. SNPs co-associated with frailty and insomnia were enriched in 9 brain regions, primarily in the cerebellum and cerebellar hemispheres (Figure 5; Supplementary Table S8).

### 3.7 Identification of shared functional genes

**Figure 6:**
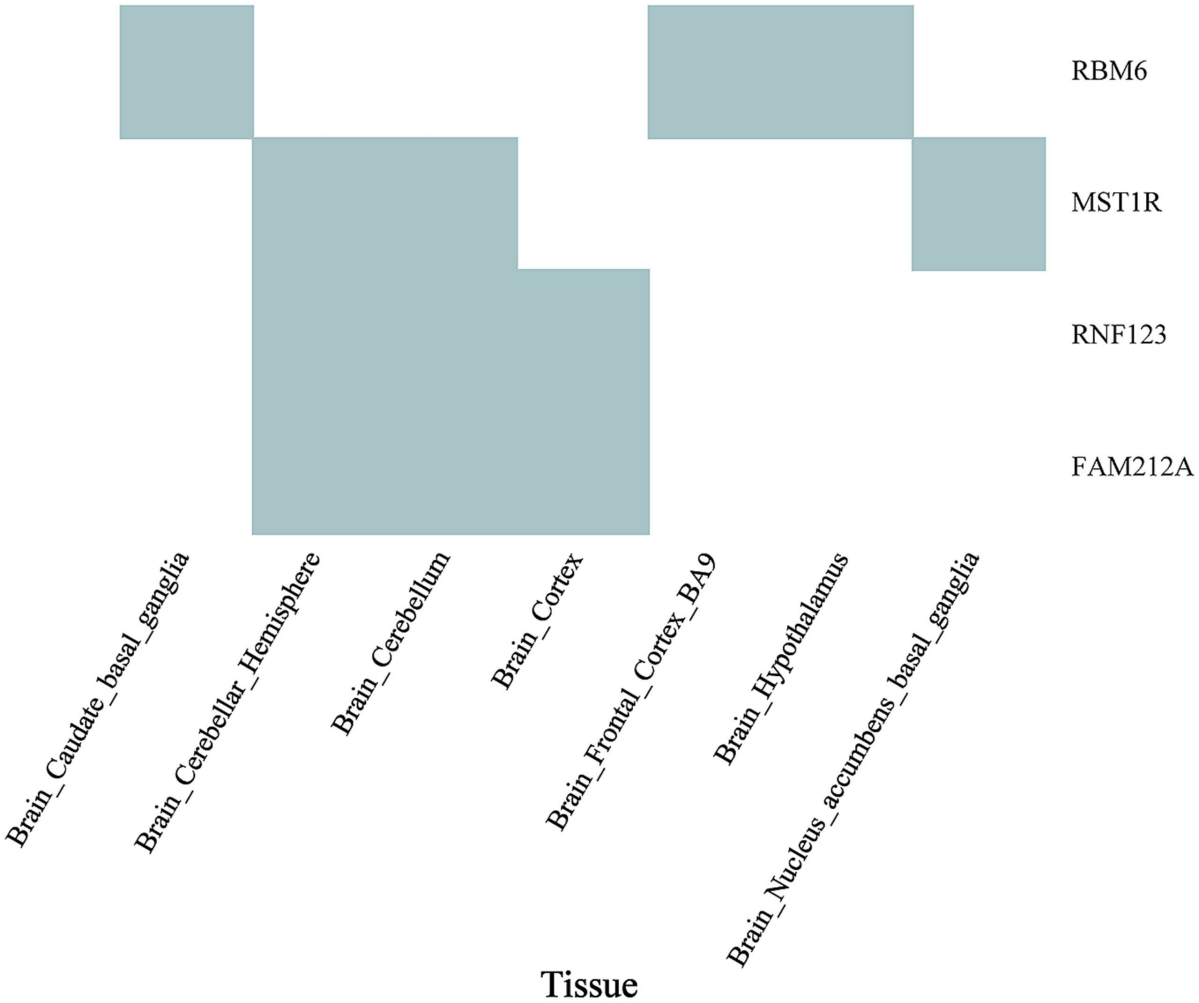
Overlap of significant genes associated with both frailty and insomnia in SMR in enriched tissues. Cyan plots indicate significant genes that met the FDR threshold of <5% in SMR analyses with GWAS for both frailty and insomnia, and also cleared the HEIDI-outlier test (*p* > 0.05).

SMR was utilized to pinpoint shared functional genes related to frailty and insomnia by jointly analyzing GWAS summary data for these conditions and eQTL summary data from GTEx. The S-LDSC and MAGMA analyses indicated associations with enriched SNP heritability in both diseases. Among these genes, four were shared between weakness and insomnia following HEIDI outlier testing. RMB6 was detected in various brain regions including the caudate basal ganglia (*p*SMR = 1.34E-06, *p*HEIDI = 0.99; *p*SMR = 2.99E-07, *p*HEIDI = 0.05), the frontal cortex (BA9) (*p*SMR = 1.28E-06, *p*HEIDI = 0.74; *p*SMR = 3.03E-07, *p*HEIDI = 0.07), and the cerebral hypothalamus (*p*SMR = 4.31E-06, *p*HEIDI = 0.74; *p*SMR = 1.26E-06, *p*HEIDI = 0.09). MST1R was identified in the cerebellar hemisphere (*p*SMR = 9.11E-06, *p*HEIDI = 0.58; *p*SMR = 4.50E-06, *p*HEIDI = 0.15), cerebellum (*p*SMR = 2.49E-06, *p*HEIDI = 0.75; *p*SMR = 6.30E-07, *p*HEIDI = 0.12), and the basal ganglia of the nucleus ambiguus (*p*SMR = 3.35E-05, *p*HEIDI = 0.95; *p*SMR = 2.14E-05, *p*HEIDI = 0.40). RF123 and FAM212A were detected in the cerebellar hemisphere, cerebellum, and cerebral hemisphere (Figure 6; Supplementary Table S9).

## 4 Discussion

To our knowledge, this study is the first genome-wide cross-trait analysis to systematically assess the shared genetic foundations of frailty and insomnia. Our research, leveraging a detailed GWAS dataset along with tissue-specific expression data, reveals potential genetic connections between frailty and insomnia. This is supported by several critical observations: Initially, we found that specific genomic regions and functional elements are linked to both frailty and insomnia. Second, bidirectional MR analyses indicate that frailty exerts a causal effect on insomnia, albeit not reciprocally. Finally, our investigation into genetic commonalities between frailty and insomnia, particularly in brain tissue, led to the identification of numerous potential functional genes impacting both phenotypes.

A path-analysis study revealed that poor sleep quality contributes to increased frailty susceptibility in older adults, potentially linked to poor physical functioning (Tang et al., 2021), and indicated that over half of pre-frail and frail older adults exhibit insomnia symptoms (Nemoto et al., 2021). The association between insomnia and aging is believed to initiate a chronic low-grade inflammatory state in older adults, leading to alterations in the immune system, a phenomenon termed inflammatory aging (Besedovsky et al., 2019; Irwin, 2019). Transitioning from a state of homeostasis to low-grade inflammation induces an inflammatory response, the severity of which is predictive of mortality in older adults (Ingiosi et al., 2013). Prolonged activation of a low-grade inflammatory state may impede the return to homeostasis during sleep repair, potentially leading to disease in older adults (Pak et al., 2020). Furthermore, extended sleep latency is known to elevate insulin resistance via the activation of inflammatory markers (Kim et al., 2016), thereby exacerbating protein synthesis impairment and muscle loss, potentially worsening limb movement.

To date, there has been no comprehensive analysis of the genetic link between frailty and insomnia in any study. Following a local genetic correlation analysis, the independent region 3p21.31 (chr3:47727212 … 0.49316972) was identified. One study reported a correlation between 3p21.31 and the incidence of gliomas and glioblastomas in women (Ostrom et al., 2018). Additionally, the TMIE gene located at 3p21.31 is believed to be significantly associated with sleep duration (Meng et al., 2022). Considering the notable gene-level correlations, a cross-trait GWAS analysis was conducted to identify 124 SNPs co-associated with frailty and insomnia, including two SNPs at 3p21.31: rs34290943 and rs10865954. The SNP rs34290943 was mapped to the DALRD3 gene, while rs10865954 was located in the MIR191 gene locus. The protein encoded by the DALRD3 gene possesses a DALR anticodon binding domain, akin to that found in the leucyl aminoacyl tRNA synthetase class. It has been shown that the expression level of DALRD3 in the brain tissue of patients with insomnia is significantly lower compared to controls, and significant dynamic changes in the expression level of this gene were observed in the mouse cerebral cortex during sleep and sleep deprivation states (Sun et al., 2020). Previous studies have suggested that significantly reduced plasma levels of MIR191 in elderly patients are associated with age-related frailty syndrome, with its notable age-related downregulation in plasma aligning with prior observations in monocytes (Serna et al., 2012). Furthermore, miR-191 is a component of a distinctive circulating 7-miRNA signature that differentiates Alzheimer’s disease patients from normal controls (Nagpal and Kulshreshtha, 2014).

Subsequent analyses of functional components uncovered 24 annotated regions linked to frailty and insomnia. It is suggested that the Coding_UCSCL2_0 region may have associations with neuropsychiatric disorders including Alzheimer’s disease, major depression, bipolar disorder, and sporadic Creutzfeldt-Jakob disease (de Souza et al., 2010). In the study by Hnisz et al. mutations in MAFbin1L2_0 were found to be associated with Alzheimer’s disease (Hnisz et al., 2013). Animal experiments demonstrated that increased H3K27ac enrichment in early gene promoters of neurons enhances memory capacity in mice, thereby influencing aging (Li et al., 2021). Intron_UCSCL2_0 is crucial for forebrain inhibitory neuronal differentiation and exhibits a strong association with restless legs syndrome (RLS) and insomnia (Lam et al., 2022). These functional components linked to frailty and insomnia require additional experimental validation.

We investigated the causal relationship between frailty and insomnia using a GSMR approach, an extension of the SMR approach that utilizes all SNPs of the highest genome-wide significance levels associated with the exposure as IVs for causal testing. The GSMR method excelled in efficacy for discovering causal effects, surpassing methods such as IVW and MR-Egger. It also demonstrated considerable robustness in the face of pleiotropy in the instrumental variables and linkage disequilibrium among instrumental variables (Zhu et al., 2018). Two-sample Mendelian randomization analyses disclosed a causal effect of frailty on insomnia and indicated horizontal pleiotropy in the causal effect of insomnia on frailty. This implies that older frail patients are more prone to insomnia symptoms, that the onset of insomnia leading to frailty could be impacted by other confounders, and that a shared genetic risk exists between frailty and insomnia. One meta-analysis suggested that insomnia independently correlates with frailty among older adults in the community or nursing homes; however, the inclusion of six studies using the FP to identify frailty partly accounts for the inconsistency in findings (Wen et al., 2023). Compared to randomized controlled trials (RCTs), Mendelian randomization offers the benefits of lower cost, diminished confounding, and lessened reverse causal inference, thus serving as a ‘natural’ RCT that mitigates traditional observational study biases and is superior in deducing causality (Emdin et al., 2017).

Tissue enrichment analysis for key genes associated with frailty and insomnia was conducted, revealing significant enrichment in brain tissues for both conditions. S-LDSC analysis demonstrated that SNPs co-associated with frailty and insomnia were enriched in 11 brain regions, mainly in the anterior cingulate cortex (BA24) and the amygdala. Similarly, MAGMA tissue-specificity analyses indicated enrichment in 9 brain regions, especially in the cerebellum and cerebellar hemispheres. This enrichment, particularly in the cerebellum and cerebellar hemispheres, suggests a potential mechanism for the co-morbidity of frailty and insomnia in brain structures. Aligned with our findings, a genome-wide analysis of insomnia (involving 593,724 cases and 1,771,286 controls) demonstrated that genes linked to insomnia were primarily enriched in brain tissues [including cerebellar hemispheres, cerebellum, frontal cortex (BA9), cerebral cortex, anterior cingulate cortex (BA24)] (Watanabe et al., 2022). A GWAS study with 386,565 participants of European ancestry from the UK Biobank revealed that genes related to frailty were predominantly enriched in specific brain areas (cerebellar hemispheres, frontal cortex BA9, cerebellum, anterior cingulate cortex BA24, nucleus ambiguus in the basal ganglia) (Ye et al., 2023). Neuroimaging meta-analyses on insomnia have suggested a critical role for the anterior cingulate cortex and amygdala in insomnia disorders (Reimann et al., 2023). Voxel-based studies have revealed that frailty is associated with multiple brain regions, potentially leading to reduced grey matter volume in areas such as the hippocampus, amygdala, fusiform gyrus, and several cortical regions (Nishita et al., 2019). Additionally, reduced grey matter in the cerebellum is recognized as a neurological feature of frailty (Chen et al., 2015). Through SMR exploration of functional genes linked to frailty and insomnia in specific enriched tissues, four genes were identified: RMB6, MST1R, RF123, and FAM212A. In line with our study, MST1R and RF123 are considered to be associated with brain function or psychiatric disorders, exhibiting upregulated expression in various brain regions (Qiu et al., 2022). The role of these identified genes in frailty and insomnia necessitates further experimental verification.

Our study presents several limitations. Firstly, the genetic data on frailty and insomnia in this study originated exclusively from European populations, potentially diminishing the heterogeneity of the results to some extent. However, this aspect also restricts the generalization of our results to other ethnic populations, and therefore, future analyses should incorporate data from diverse ethnicities. Secondly, the genetic data encompassed a large cohort of patients with insomnia symptoms, thereby enlarging the sample size of the data, but also introduced a certain level of heterogeneity in the findings. Incorporating additional GWAS data that integrate genetic information with comprehensive clinical characteristics (e.g., varying severities of insomnia, duration of symptoms, frequency of occurrence) in subsequent studies may yield a more nuanced comprehension of the role genetic factors play in the varied clinical manifestations of frailty and insomnia. Thirdly, our analyses were confined to the genetic characteristics of the traits, acknowledging that epigenetic influences on these traits are equally crucial.

## 5 Conclusion

In conclusion, our research revealed a notable genetic link between frailty and insomnia, highlighted by the identification of shared risk SNPs. This correlation was found to be more distinct in certain genomic areas. Partitioned genetic analyses revealed 24 functional elements significantly linked to both frailty and insomnia. Mendelian randomization was additionally employed to demonstrate a causal relationship between frailty and insomnia. The genetic correlation was found to be enriched in 11/9 brain regions, within which 4 functional genes were detected. These findings may offer insights into the shared genetic architecture of frailty and insomnia, contributing to an enhanced understanding of their pathogenesis and the development of therapeutic targets aimed at alleviating insomnia symptoms and potentially preventing or reversing aging.

## Data availability statement

Publicly available datasets were analyzed in this study. Data about the frailty index can be found at: https://www.ebi.ac.uk/gwas/downloads/summary-statistics GCST90020053. Data about insomnia can be found at https://gwas.mrcieu.ac.uk/datasets/ukb-b-3957.

## Ethics statement

Written informed consent was not required for any of the potentially identifiable images or data included in this study, as all statistical analyses were based on existing publicly available summary data and thus no additional ethical approval was required.

## Author contributions

ZS: Visualization, Writing – review & editing. WL: Formal analysis, Writing – original draft. YH: Formal analysis, Writing – original draft. YX: Data curation, Writing – original draft. YW: Supervision, Writing – review & editing.
