# Supplementary material for: Investigating the shared genetic architecture between frailty and insomnia
Source: Front Aging Neurosci. 2024 Feb 15;16:1358996. doi: 10.3389/fnagi.2024.1358996 (PMC10903740; doi:10.3389/fnagi.2024.1358996)
Supplement: Supplementary file 1 [file Data_Sheet_1.docx]

**Supplemental Table S1.** **Genetic correlations between** **frailty and insomnia.**

| **With constrained intercept of heritability** | | | | | | |
| --- | --- | --- | --- | --- | --- | --- |
| **r_g_(SE)** | ***P*-value** | **Genetic covariance (SE)** | **Frailty**  **Lambda GC** | **Frailty**  **Intercept** | **insomnia**  **Lambda GC** | **insomnia**  **Intercept** |
| 0.6121  (0.0209) | 4.6302e-189 | 0.0506 (0.0025) | 1.3238 | 1.0197 (0.0087) | 1.4926 | 1.0267 (0.0092) |
| **Without constrained intercept** | | | | | | |
| **r_g_(SE)** | ***P*-value** | **Genetic covariance (SE)** | **Frailty**  **Lambda GC** | **Frailty**  **Intercept** | **insomnia**  **Lambda GC** | **insomnia**  **Intercept** |
| 1.1429 (0.0191) | 0.0 | 0.1008 (0.0019) | 1.3238 | constrained to 1 | 1.4926 | constrained to 1 |

SE: standard error.

**Supplementary Table S2. Summary of local genetic correlations between frailty and insomnia.**

| **Chr** | **Start** | **End** | **Number of SNPs** | **local_rhog** | **SE** | ***P*** |
| --- | --- | --- | --- | --- | --- | --- |
| 3 | 47727212 | 49316972 | 967 | 0.00028557 | 6.45E-05 | 9.45E-06 |
| SE: standard error; SNP: single nucleotide polymorphisms. The start and end described the base pair. | | | | | | |

**Supplementary Table S3. Partitioned heritability between frailty and insomnia.**

| **Category** | **disease** | **Prop._h2** | **Prop._h2_std_error** | **Enrichment** | **Enrichment_std_error** | **Enrichment_p** |
| --- | --- | --- | --- | --- | --- | --- |
| GERP.RSsup4L2_0 | frailty | 0.104691309 | 0.031249368 | 12.845677306 | 3.834313469 | 0.001827541 |
|  | insomnia | 0.135044985 | 0.033272524 | 16.57008887 | 4.082555803 | 0.000200596 |
| Coding_UCSCL2_0 | frailty | 0.065502159 | 0.021728667 | 4.593696352 | 1.523841375 | 0.018524734 |
|  | insomnia | 0.245275888 | 0.045993973 | 12.728993494 | 2.386932482 | 4.03824E-06 |
| BLUEPRINT_DNA_methylation_MaxCPPL2_0 | frailty | 0.089425595 | 0.020928032 | 2.818243204 | 0.659545903 | 0.007476554 |
|  | insomnia | 0.207796873 | 0.042860386 | 9.692264736 | 1.999136007 | 2.55374E-05 |
| GERP.NSL2_0 | frailty | 3.378232611 | 0.177829401 | 1.935599121 | 0.1018895 | 3.30911E-18 |
|  | insomnia | 0.196277747 | 0.041467108 | 6.667936717 | 1.408718298 | 8.9205E-05 |
| H3K4me3_TrynkaL2_0 | frailty | 0.235361115 | 0.049374457 | 1.771140681 | 0.371552916 | 0.037973149 |
|  | insomnia | 0.078187278 | 0.020413588 | 5.770543474 | 1.506606976 | 0.001576022 |
| MAFbin10L2_0 | frailty | 0.169388915 | 0.023377191 | 1.720338245 | 0.23742212 | 0.002161536 |
|  | insomnia | 0.135914047 | 0.041685792 | 5.509164288 | 1.689699336 | 0.008634594 |
| SuperEnhancer_HniszL2_0 | frailty | 0.238696815 | 0.01932863 | 1.427554933 | 0.115597187 | 0.00033925 |
|  | insomnia | 0.059447585 | 0.013882494 | 3.906654139 | 0.912301187 | 0.001575729 |
| H3K27ac_HniszL2_0 | frailty | 0.485687153 | 0.032946641 | 1.248412498 | 0.084686197 | 0.004087765 |
|  | insomnia | 0.055339724 | 0.016760224 | 3.881000165 | 1.17540217 | 0.014712136 |
| Backgrd_Selection_StatL2_0 | frailty | 0.218358567 | 0.007101106 | 1.228667558 | 0.039956752 | 3.71843E-10 |
|  | insomnia | 0.059651076 | 0.020088443 | 3.349878 | 1.128124368 | 0.035848137 |
| Intron_UCSCL2_0 | frailty | 0.457506902 | 0.023588426 | 1.180811273 | 0.060881005 | 0.003215199 |
|  | insomnia | 0.03378655 | 0.011313529 | 3.267428621 | 1.09410845 | 0.03881474 |
| CpG_Content_50kbL2_0 | frailty | 0.01090063 | 0.000187881 | 1.084437646 | 0.01869115 | 2.80887E-05 |
|  | insomnia | 0.082860564 | 0.01548298 | 2.611346583 | 0.487945352 | 0.001327836 |
| Conserved_Primate_phastCons46wayL2_0 | frailty | 0.228040633 | 0.040902295 | 11.834541761 | 2.122691543 | 5.5569E-07 |
|  | insomnia | 3.356814712 | 0.160883481 | 1.923327477 | 0.092180131 | 2.5174E-18 |
| Nucleotide_Diversity_10kbL2_0 | frailty | 3.762701535 | 0.110973559 | 0.816435294 | 0.02407917 | 1.34802E-13 |
|  | insomnia | 0.252745615 | 0.041040733 | 1.901962609 | 0.308839934 | 0.003579568 |
| MAFbin1L2_0 | frailty | 0.065575269 | 0.015626233 | 0.640650783 | 0.152663622 | 0.01603742 |
|  | insomnia | 0.144314495 | 0.01688844 | 1.465678825 | 0.171521429 | 0.006412886 |
| MAFbin2L2_0 | frailty | 0.021297098 | 0.018612752 | 0.213279355 | 0.186397021 | 1.77735E-05 |
|  | insomnia | 0.219037729 | 0.015681136 | 1.309981412 | 0.093782916 | 0.001284674 |
| Conserved_Mammal_phastCons46wayL2_0 | frailty | 0.213982174 | 0.045530883 | 9.980765566 | 2.123695919 | 2.41508E-05 |
|  | insomnia | 0.453012878 | 0.026352455 | 1.164426391 | 0.067736471 | 0.016574017 |
| Conserved_Vertebrate_phastCons46wayL2_0 | frailty | 0.23097408 | 0.045982164 | 7.846638598 | 1.56210351 | 1.23241E-05 |
|  | insomnia | 0.010843412 | 0.000156382 | 1.078745368 | 0.015557452 | 2.41095E-06 |
| Conserved_LindbladTohL2_0 | frailty | 0.188577213 | 0.044787781 | 7.643822449 | 1.815435913 | 0.00035027 |
|  | insomnia | 3.789219735 | 0.10023586 | 0.822189243 | 0.021749292 | 9.37735E-15 |
| BivFlnkL2_0 | frailty | 0.097353604 | 0.028125463 | 7.185097322 | 2.07577514 | 0.002779301 |
|  | insomnia | 0.055377332 | 0.012227671 | 0.541019987 | 0.119460691 | 8.16361E-05 |
| GTEx_eQTL_MaxCPPL2_0 | frailty | 0.059442705 | 0.014672041 | 5.748583322 | 1.418903303 | 0.000864555 |
|  | insomnia | 0.041734054 | 0.01353622 | 0.417944829 | 0.135558198 | 1.55177E-05 |

**Supplemental Table S4. Genome-wide significant loci in genome-wide cross-trait analysis methods.**

| **SNP** | **CHR** | **BP** | **Effect Allele** | **Non-**  **effect Allele** | **mtag_pval_**  **frailty** | **mtag_pval_**  **insomnia** | **Cpassoc Pval** |
| --- | --- | --- | --- | --- | --- | --- | --- |
| rs10038581 | 5 | 104052400 | C | T | 4.80E-11 | 4.30E-11 | 1.75E-10 |
| rs1032524 | 1 | 201790826 | C | T | 2.22E-14 | 1.60E-14 | 2.05E-15 |
| rs1049053 | 6 | 32634405 | T | C | 2.19E-11 | 4.15E-11 | 1.46E-18 |
| rs10865954 | 3 | 49211989 | C | T | 4.73E-12 | 3.53E-12 | 5.38E-13 |
| rs11079993 | 17 | 50301552 | T | G | 2.34E-10 | 2.47E-10 | 4.72E-08 |
| rs11152363 | 18 | 53057188 | A | G | 7.61E-13 | 4.29E-13 | 5.33E-16 |
| rs11191454 | 10 | 104660004 | G | A | 3.03E-09 | 2.19E-09 | 9.24E-11 |
| rs11203766 | 8 | 12571233 | G | A | 2.71E-08 | 2.19E-08 | 6.99E-09 |
| rs113315602 | 6 | 32574575 | C | A | 6.78E-09 | 1.26E-08 | 9.95E-18 |
| rs113851554 | 2 | 66750564 | T | G | 6.79E-30 | 1.11E-30 | 4.07E-46 |
| rs1142338 | 6 | 32609322 | T | C | 2.46E-09 | 4.17E-09 | 2.63E-15 |
| rs114281696 | 6 | 32525925 | G | A | 1.91E-08 | 3.09E-08 | 4.94E-14 |
| rs116139747 | 3 | 53556816 | C | A | 1.76E-09 | 1.65E-09 | 1.07E-08 |
| rs11631027 | 15 | 96967788 | C | A | 6.86E-09 | 6.39E-09 | 3.38E-08 |
| rs11636381 | 15 | 74134883 | C | T | 6.48E-09 | 4.75E-09 | 2.55E-10 |
| rs11660554 | 18 | 52796510 | A | G | 3.87E-11 | 4.02E-11 | 7.13E-09 |
| rs11675817 | 2 | 59169043 | A | G | 8.72E-09 | 6.98E-09 | 3.96E-09 |
| rs11752277 | 6 | 32537771 | G | A | 1.02E-08 | 1.38E-08 | 8.56E-10 |
| rs11790060 | 9 | 96202932 | C | T | 2.51E-12 | 1.90E-12 | 4.03E-13 |
| rs12424599 | 12 | 109866615 | G | A | 2.01E-13 | 1.77E-13 | 8.49E-13 |
| rs12477304 | 2 | 200970371 | G | T | 4.88E-09 | 3.93E-09 | 1.43E-09 |
| rs12524473 | 6 | 14105768 | G | A | 6.22E-09 | 5.78E-09 | 2.95E-08 |
| rs12536335 | 7 | 114043159 | G | A | 5.33E-15 | 4.51E-15 | 1.34E-14 |
| rs12671330 | 7 | 114334043 | C | T | 6.23E-15 | 6.81E-15 | 1.85E-12 |
| rs1281934 | 6 | 32584382 | G | A | 1.24E-09 | 2.19E-09 | 2.26E-16 |
| rs12936234 | 17 | 43177951 | C | T | 3.39E-11 | 2.60E-11 | 5.93E-12 |
| rs13229777 | 7 | 52581406 | G | A | 1.72E-09 | 1.48E-09 | 1.90E-09 |
| rs143286107 | 2 | 210050593 | C | A | 3.81E-09 | 3.67E-09 | 3.67E-08 |
| rs145244672 | 6 | 32556461 | G | T | 4.20E-09 | 7.84E-09 | 5.71E-18 |
| rs1456029 | 7 | 114290748 | G | A | 5.06E-11 | 4.59E-11 | 2.32E-10 |
| rs146549424 | 18 | 42676081 | C | T | 3.43E-08 | 3.02E-08 | 4.79E-08 |
| rs1499761 | 5 | 135753619 | G | A | 5.62E-09 | 4.52E-09 | 1.54E-09 |
| rs1547631 | 13 | 112643785 | C | A | 2.14E-08 | 1.65E-08 | 2.12E-09 |
| rs1660237 | 18 | 53013947 | T | C | 8.36E-10 | 6.80E-10 | 7.38E-10 |
| rs17312861 | 7 | 114125510 | G | A | 3.46E-08 | 3.05E-08 | 4.89E-08 |
| rs1731947 | 7 | 137081525 | G | A | 4.59E-09 | 4.21E-09 | 1.74E-08 |
| rs17425622 | 6 | 32571961 | C | T | 2.54E-12 | 5.67E-12 | 5.10E-23 |
| rs17459384 | 1 | 57870215 | G | A | 6.97E-11 | 4.83E-11 | 1.31E-12 |
| rs17550821 | 2 | 58978523 | A | G | 1.20E-08 | 7.02E-09 | 1.12E-13 |
| rs17612518 | 6 | 32614927 | T | G | 1.13E-09 | 2.20E-09 | 3.80E-19 |
| rs176644 | 15 | 89913632 | T | G | 2.61E-08 | 2.15E-08 | 1.87E-08 |
| rs1909650 | 13 | 53791895 | A | G | 2.97E-18 | 2.31E-18 | 1.53E-17 |
| rs1942262 | 18 | 52873317 | A | G | 3.55E-13 | 2.36E-13 | 1.15E-14 |
| rs1988337 | 4 | 91292200 | G | A | 2.09E-11 | 1.82E-11 | 4.94E-11 |
| rs2062113 | 16 | 59476179 | C | T | 1.19E-09 | 8.54E-10 | 3.59E-11 |
| rs2073167 | 22 | 41791536 | C | T | 5.63E-09 | 5.36E-09 | 4.24E-08 |
| rs2155292 | 11 | 112910783 | G | A | 1.51E-12 | 1.64E-12 | 2.57E-10 |
| rs2194662 | 2 | 67087305 | C | A | 1.47E-09 | 1.47E-09 | 3.29E-08 |
| rs2242656 | 6 | 31614102 | T | C | 3.72E-10 | 5.16E-10 | 6.68E-11 |
| rs2604551 | 4 | 15091201 | G | T | 2.30E-10 | 1.98E-10 | 3.38E-10 |
| rs2604853 | 3 | 35520338 | C | T | 2.77E-09 | 2.76E-09 | 4.90E-08 |
| rs2815764 | 1 | 72754123 | G | A | 2.77E-09 | 2.61E-09 | 1.86E-08 |
| rs2838788 | 21 | 46542374 | C | T | 6.18E-09 | 5.56E-09 | 1.52E-08 |
| rs28528997 | 7 | 2074122 | G | A | 1.56E-09 | 1.48E-09 | 1.32E-08 |
| rs2956287 | 12 | 84709216 | G | A | 5.38E-10 | 4.18E-10 | 9.50E-11 |
| rs3091280 | 6 | 31586742 | A | G | 1.18E-09 | 1.72E-09 | 8.81E-12 |
| rs3129306 | 6 | 32953982 | A | G | 6.63E-10 | 9.70E-10 | 5.80E-12 |
| rs314280 | 6 | 105400837 | G | A | 6.17E-10 | 4.38E-10 | 1.55E-11 |
| rs323509 | 5 | 104082179 | C | A | 1.54E-15 | 1.31E-15 | 5.21E-15 |
| rs324017 | 12 | 57487814 | C | A | 5.23E-12 | 4.30E-12 | 4.15E-12 |
| rs34290943 | 3 | 49161660 | C | T | 5.81E-10 | 5.63E-10 | 7.55E-09 |
| rs34356918 | 17 | 43169715 | A | G | 3.13E-08 | 2.45E-08 | 6.99E-09 |
| rs34476507 | 2 | 200651678 | C | T | 2.29E-09 | 2.09E-09 | 8.04E-09 |
| rs35021449 | 6 | 32530583 | T | C | 7.46E-10 | 1.30E-09 | 3.20E-16 |
| rs35096827 | 3 | 150616348 | T | C | 6.07E-11 | 6.78E-11 | 3.89E-08 |
| rs35198836 | 16 | 1244631 | T | C | 2.32E-08 | 1.90E-08 | 1.54E-08 |
| rs35267450 | 1 | 18429405 | C | T | 2.29E-08 | 1.85E-08 | 6.15E-09 |
| rs35881094 | 2 | 58922921 | G | T | 1.23E-09 | 7.09E-10 | 2.61E-14 |
| rs36116812 | 5 | 141277172 | C | T | 1.78E-08 | 1.49E-08 | 9.62E-09 |
| rs36213483 | 6 | 32611997 | T | C | 3.37E-14 | 7.54E-14 | 2.84E-23 |
| rs4572538 | 2 | 147423012 | T | C | 7.26E-09 | 5.32E-09 | 4.70E-10 |
| rs4577309 | 2 | 191288833 | G | A | 2.34E-09 | 1.86E-09 | 5.68E-10 |
| rs4723649 | 7 | 1071905 | C | T | 9.09E-11 | 7.24E-11 | 3.01E-11 |
| rs4886140 | 13 | 59833519 | G | A | 2.85E-14 | 2.22E-14 | 1.19E-14 |
| rs4886867 | 15 | 74348374 | A | G | 2.05E-09 | 1.40E-09 | 3.36E-11 |
| rs518143 | 11 | 83335764 | G | A | 1.09E-10 | 1.02E-10 | 8.14E-10 |
| rs521956 | 3 | 173117975 | C | T | 6.37E-10 | 8.09E-10 | 4.36E-09 |
| rs529480034 | 6 | 32619229 | T | C | 5.75E-09 | 8.62E-09 | 3.34E-12 |
| rs533757 | 11 | 107397587 | C | T | 2.51E-08 | 2.13E-08 | 1.78E-08 |
| rs555911977 | 6 | 32535454 | T | C | 1.79E-08 | 2.82E-08 | 3.04E-13 |
| rs55892967 | 2 | 66983321 | C | T | 1.33E-08 | 1.02E-08 | 1.31E-09 |
| rs56093896 | 2 | 114103966 | A | C | 1.20E-08 | 7.81E-09 | 1.80E-11 |
| rs56254970 | 10 | 104219293 | G | A | 3.82E-09 | 2.82E-09 | 1.88E-10 |
| rs575147125 | 6 | 32619144 | G | T | 4.63E-10 | 8.86E-10 | 1.11E-18 |
| rs6058750 | 20 | 31177986 | C | T | 1.84E-11 | 1.74E-11 | 1.95E-10 |
| rs60751869 | 2 | 66816983 | T | G | 3.28E-10 | 2.12E-10 | 1.88E-12 |
| rs624244 | 18 | 53183396 | A | G | 1.75E-08 | 1.45E-08 | 1.46E-08 |
| rs6452792 | 5 | 87792844 | A | G | 6.14E-11 | 4.89E-11 | 5.14E-11 |
| rs647983 | 5 | 88048071 | C | T | 1.83E-09 | 1.54E-09 | 1.38E-09 |
| rs6503430 | 17 | 43278732 | T | C | 1.57E-10 | 1.24E-10 | 9.74E-11 |
| rs66791238 | 18 | 53230650 | C | T | 3.72E-09 | 3.06E-09 | 1.72E-09 |
| rs6719650 | 2 | 164550641 | G | A | 1.94E-08 | 1.73E-08 | 3.81E-08 |
| rs6759659 | 2 | 44143756 | G | A | 6.04E-11 | 6.30E-11 | 3.48E-09 |
| rs6809879 | 3 | 49834571 | G | A | 1.67E-11 | 1.65E-11 | 3.99E-10 |
| rs6910879 | 6 | 32560739 | G | A | 3.09E-12 | 5.84E-12 | 1.22E-18 |
| rs6938026 | 6 | 43185733 | G | A | 8.09E-10 | 6.92E-10 | 9.16E-10 |
| rs7173565 | 15 | 38850330 | C | T | 1.58E-09 | 1.51E-09 | 1.43E-08 |
| rs72745452 | 15 | 67628221 | G | T | 1.38E-11 | 1.20E-11 | 3.49E-11 |
| rs73077157 | 3 | 49749705 | C | A | 3.18E-09 | 2.92E-09 | 1.30E-08 |
| rs743581 | 15 | 74328141 | T | G | 2.25E-08 | 1.74E-08 | 4.21E-09 |
| rs7497457 | 15 | 68132723 | A | C | 2.83E-10 | 2.35E-10 | 4.59E-10 |
| rs75146758 | 16 | 50231209 | G | T | 4.37E-10 | 4.42E-10 | 1.26E-08 |
| rs7568599 | 2 | 53028060 | C | T | 4.09E-10 | 3.81E-10 | 2.61E-09 |
| rs7606845 | 2 | 215509925 | C | A | 3.73E-10 | 3.59E-10 | 4.27E-09 |
| rs7625896 | 3 | 44062561 | G | A | 1.05E-10 | 9.58E-11 | 4.72E-10 |
| rs76265753 | 1 | 161181134 | T | C | 4.14E-08 | 3.40E-08 | 2.55E-08 |
| rs7658313 | 4 | 82285904 | C | T | 3.23E-08 | 2.78E-08 | 3.03E-08 |
| rs7691487 | 4 | 90832001 | C | T | 1.87E-09 | 1.65E-09 | 3.35E-09 |
| rs7731706 | 5 | 135570987 | G | T | 1.00E-09 | 8.98E-10 | 2.70E-09 |
| rs7734045 | 5 | 135538341 | C | A | 6.29E-12 | 4.44E-12 | 2.33E-13 |
| rs78206187 | 2 | 67022234 | G | A | 1.21E-13 | 8.67E-14 | 8.89E-15 |
| rs79780963 | 10 | 104952499 | T | C | 8.48E-09 | 6.19E-09 | 4.98E-10 |
| rs801733 | 11 | 65934549 | C | A | 2.31E-09 | 1.87E-09 | 8.09E-10 |
| rs8067974 | 17 | 43232566 | G | A | 2.42E-20 | 1.73E-20 | 5.59E-21 |
| rs817509 | 3 | 117750861 | G | A | 1.23E-08 | 9.68E-09 | 2.04E-09 |
| rs833785 | 2 | 36220820 | G | T | 1.87E-09 | 1.82E-09 | 2.40E-08 |
| rs9268557 | 6 | 32389305 | C | T | 1.98E-10 | 2.91E-10 | 2.97E-12 |
| rs9268848 | 6 | 32429303 | A | G | 3.80E-10 | 5.77E-10 | 7.55E-13 |
| rs9273505 | 6 | 32628420 | T | C | 2.87E-14 | 7.88E-14 | 9.05E-29 |
| rs9404054 | 6 | 101256597 | G | A | 1.69E-10 | 1.48E-10 | 3.24E-10 |
| rs9536347 | 13 | 53644011 | T | C | 1.18E-09 | 9.23E-10 | 4.72E-10 |
| rs959301 | 8 | 107790203 | G | A | 5.38E-09 | 4.76E-09 | 9.95E-09 |
| rs9845387 | 3 | 116425935 | A | C | 1.99E-09 | 1.57E-09 | 8.34E-10 |
| rs9906181 | 17 | 21297686 | G | A | 4.93E-08 | 3.86E-08 | 6.30E-09 |

CHR: chromosome; Pval: *P* value; SNP: single-nucleotide polymorphism.

**Supplementary Table S5. Mendelian Randomization (MR) analyses between frailty and insomnia.**

| **method** | **nsnp** | **b** | **se** | **pval** |
| --- | --- | --- | --- | --- |
| MR Egger | 68 | 0.0483159 | 0.0548987 | 0.3820033 |
| Weighted median | 68 | 0.0990444 | 0.0174046 | 0 |
| Inverse variance weighted | 68 | 0.1337054 | 0.0164205 | 0 |
| Simple mode | 68 | 0.0958338 | 0.0423268 | 0.0268073 |
| Weighted mode | 68 | 0.0868904 | 0.0343522 | 0.0137851 |

**Supplementary Table S6. Mendelian Randomization (MR) analyses between insomnia and frailty.**

| **method** | **nsnp** | **b** | **se** | **pval** |
| --- | --- | --- | --- | --- |
| MR Egger | 141 | 0.2758357 | 0.1559835 | 0.0791936 |
| Weighted median | 141 | 0.6005073 | 0.0573456 | 0 |
| Inverse variance weighted | 141 | 0.6876755 | 0.0453481 | 0 |
| Simple mode | 141 | 0.6455561 | 0.1757642 | 0.0003405 |
| Weighted mode | 141 | 0.5465795 | 0.138831 | 0.0001295 |

**Supplementary Table S7. Tissue specific enrichment of SNP heritability for frailty and insomnia through S-LDSC.**

| **Tissue Type** | **disease** | **Coefficient** | **Coefficient_std_error** | **Coefficient_P_value** | **Negative_Log10_P_values** |
| --- | --- | --- | --- | --- | --- |
| Brain_Cerebellum | frailty | 4.45E-09 | 1.86E-09 | 0.008201951 | 2.086082809 |
|  | insomnia | 1.90E-09 | 7.89E-10 | 0.008055001 | 2.093934391 |
| Brain_Nucleus_accumbens_basal_ganglia | frailty | 4.19E-09 | 1.75E-09 | 0.008300354 | 2.080903411 |
|  | insomnia | 2.04E-09 | 7.74E-10 | 0.00426551 | 2.370029046 |
| Brain_Hippocampus | frailty | 4.25E-09 | 1.62E-09 | 0.004252337 | 2.3713723 |
|  | insomnia | 2.19E-09 | 7.51E-10 | 0.001746466 | 2.75783992 |
| Brain_Hypothalamus | frailty | 4.13E-09 | 1.70E-09 | 0.007452472 | 2.127699663 |
|  | insomnia | 2.31E-09 | 7.79E-10 | 0.001490725 | 2.826602355 |
| Brain_Frontal_Cortex_BA9 | frailty | 4.31E-09 | 1.62E-09 | 0.003805619 | 2.419574727 |
|  | insomnia | 2.37E-09 | 7.89E-10 | 0.00132507 | 2.87776112 |
| Brain_Cerebellar_Hemisphere | frailty | 4.79E-09 | 1.79E-09 | 0.003620547 | 2.441225757 |
|  | insomnia | 2.34E-09 | 7.68E-10 | 0.001167004 | 2.9329276 |
| Brain_Putamen_basal_ganglia | frailty | 4.52E-09 | 1.72E-09 | 0.004295489 | 2.366987428 |
|  | insomnia | 2.44E-09 | 7.87E-10 | 0.000977381 | 3.009936264 |
| Brain_Caudate_basal_ganglia | frailty | 4.41E-09 | 1.69E-09 | 0.004594038 | 2.337805443 |
|  | insomnia | 2.47E-09 | 7.92E-10 | 0.000902496 | 3.044554703 |
| Brain_Cortex | frailty | 4.20E-09 | 1.58E-09 | 0.004030705 | 2.39461902 |
|  | insomnia | 2.73E-09 | 8.09E-10 | 0.000374596 | 3.426436944 |
| Brain_Amygdala | frailty | 4.14E-09 | 1.58E-09 | 0.004314849 | 2.365034389 |
|  | insomnia | 2.89E-09 | 7.98E-10 | 0.000149459 | 3.825477489 |
| Brain_Anterior_cingulate_cortex_BA24 | frailty | 4.91E-09 | 1.54E-09 | 0.000728143 | 3.137783401 |
|  | insomnia | 2.84E-09 | 7.57E-10 | 8.67E-05 | 4.061935918 |

**Supplementary Table S8. Tissue specific enrichment of SNP heritability for frailty and insomnia through MAGMA.**

| **Tissue Type** | **Disease** | **NGENES** | **BETA** | **BETA_STD** | **SE** | **P** | **Negative_Log10_P_values** |
| --- | --- | --- | --- | --- | --- | --- | --- |
| Brain_Frontal_Cortex_BA9 | insomnia | 17285.00 | 0.04 | 0.07 | 0.01 | 0.00 | 6.15 |
|  | frailty | 17168.00 | 0.02 | 0.04 | 0.01 | 0.00 | 2.97 |
| Brain_Cortex | insomnia | 17285.00 | 0.04 | 0.07 | 0.01 | 0.00 | 5.97 |
|  | frailty | 17168.00 | 0.02 | 0.04 | 0.01 | 0.00 | 2.63 |
| Brain_Anterior_cingulate_cortex_BA24 | insomnia | 17285.00 | 0.03 | 0.06 | 0.01 | 0.00 | 4.95 |
|  | frailty | 17168.00 | 0.02 | 0.04 | 0.01 | 0.00 | 2.38 |
| Brain_Cerebellum | insomnia | 17285.00 | 0.04 | 0.07 | 0.01 | 0.00 | 7.09 |
|  | frailty | 17168.00 | 0.02 | 0.03 | 0.01 | 0.01 | 2.25 |
| Brain_Cerebellar_Hemisphere | insomnia | 17285.00 | 0.04 | 0.07 | 0.01 | 0.00 | 6.76 |
|  | frailty | 17168.00 | 0.02 | 0.03 | 0.01 | 0.01 | 2.22 |
| Brain_Hypothalamus | insomnia | 17285.00 | 0.04 | 0.07 | 0.01 | 0.00 | 5.30 |
|  | frailty | 17168.00 | 0.02 | 0.03 | 0.01 | 0.01 | 1.92 |
| Brain_Nucleus_accumbens_basal_ganglia | insomnia | 17285.00 | 0.03 | 0.05 | 0.01 | 0.00 | 3.76 |
|  | frailty | 17168.00 | 0.02 | 0.03 | 0.01 | 0.02 | 1.82 |
| Brain_Amygdala | insomnia | 17285.00 | 0.03 | 0.05 | 0.01 | 0.00 | 3.71 |
|  | frailty | 17168.00 | 0.02 | 0.03 | 0.01 | 0.02 | 1.62 |
| Brain_Caudate_basal_ganglia | insomnia | 17285.00 | 0.03 | 0.05 | 0.01 | 0.00 | 3.65 |
|  | frailty | 17168.00 | 0.01 | 0.03 | 0.01 | 0.04 | 1.36 |

**Supplementary Table S9. Overlap of significant genes associated with both frailty and insomnia in SMR in enriched tissues.**

| **Gene** | **p_SMR_frailty** | **p_HEIDI_frailty** | **p_SMR_insomnia** | **p_HEIDI_insomnia** |
| --- | --- | --- | --- | --- |
| RBM6 | 1.36E-06 | 0.9873224 | 2.99E-07 | 0.05052858 |
| FAM212A | 1.14E-05 | 0.3665996 | 4.32E-06 | 0.3041604 |
| MST1R | 9.11E-06 | 0.5758076 | 4.50E-06 | 0.1497547 |
| RNF123 | 3.58E-05 | 0.9148694 | 1.69E-05 | 0.8781917 |
| FAM212A | 4.64E-06 | 0.4461225 | 1.64E-06 | 0.126054 |
| MST1R | 2.49E-06 | 0.7543926 | 6.30E-07 | 0.1173931 |
| RNF123 | 1.03E-05 | 0.4147934 | 3.62E-06 | 0.2306435 |
| MST1R | 6.76E-05 | 0.5198542 | 3.10E-05 | 0.5408704 |
| RNF123 | 8.06E-05 | 0.4002944 | 1.87E-05 | 0.853888 |
| RBM6 | 1.28E-06 | 0.7378422 | 3.03E-07 | 0.07068191 |
| RBM6 | 4.31E-06 | 0.7393134 | 1.26E-06 | 0.08679003 |
| MST1R | 3.35E-05 | 0.9475818 | 2.14E-05 | 0.3993638 |

**
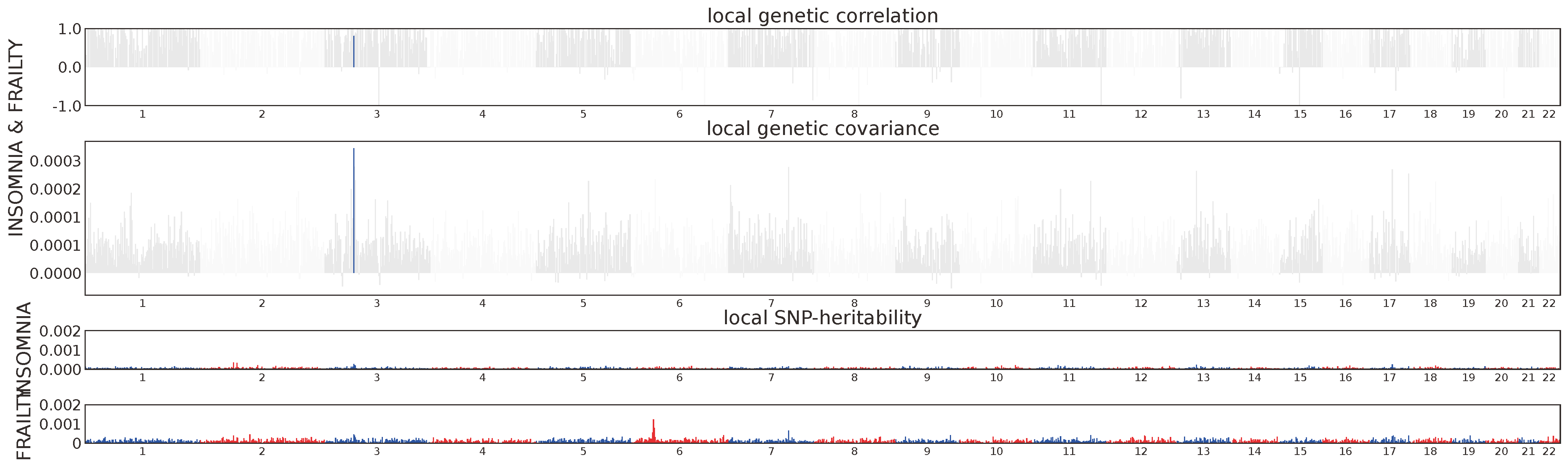
**

**Supplementary Figure1. Summary of local genetic correlations between frailty and insomnia. Local genetic correlation between frailty and insomnia.** Manhattan plot showed the estimates of local genetic correlation and local genetic covariance between frailty and insomnia, local SNP heritability of frailty and insomnia, respectively. Red and blue bars in ‘local genetic correlation’ and ‘local genetic covariance’ represent significant regions which shared SNP heritability, after multiple adjustment (P <5E−08 in both local SNP heritability test, and, P < 0.05/1703 in local genetic covariance test).
